# Indoor and outdoor noise changes due to the COVID-19 lockdown and their effects on individuals’ expectations and preferences

**DOI:** 10.1038/s41598-021-96098-w

**Published:** 2021-08-16

**Authors:** Marco Caniato, Federica Bettarello, Andrea Gasparella

**Affiliations:** 1 Faculty of Science and Technology, Free University of Bozen-Bolzano, Bozen, Italy; 2 Department of Engineering and Architecture, University of Trieste, Trieste, Italy

**Keywords:** Environmental impact, Engineering

## Abstract

The COVID-19 pandemic significantly modified our urban territories. One of the most strongly affected parameters was outdoor noise, caused by traffic and human activity in general, all of which were forced to stop during the spring of 2020. This caused an indubitable noise reduction both inside and outside the home. This study investigates how people reacted to this new unexpected, unwanted and unpredictable situation. Using field measurements, it was possible to demonstrate how the outdoor sound pressure level clearly decreased. Furthermore, by means of an international survey, it was discovered that people had positive reaction to the lower noise level. This preference was generally not related to home typology or location in the city, but rather to a generalized wish to live in a quieter urban environment.

## Introduction

During the COVID-19 emergency many different human activities have been strongly modified or banned. The pandemic has made many people around the world stay at home at some point in time, strongly limiting actions and unfortunately personal freedom. These restrictions have been necessary to save lives. There have been many consequences of this: (1) economic crisis, (2) a stop to working activities and/or smart working from home, (3) imposed distances and (4) strong reduction in traffic and normal activities. As an example, in Italy the traffic decreased by 79% during April 2020 compared to April 2019^1^. This situation affected the outdoor environment^2,3^ in terms of pollutant emissions; one of these is noise^4–6^. During this period, the outdoor sound pressure level significantly decreased^7–10^.

In Europe, the first pandemic wave started in Italy in February 2020. Soon after, this was experienced in other European countries^11–13^. Confinement and restrictions were applied almost everywhere for the first time in the modern age. Remote working became part of the daily routine for those who could do it^14–16^. Others experienced forced holidays or work suspension^17^. Schools where also closed and pupils had to attend online lessons^18–20^ and this created stress to students^21^. No outdoor sport practice was allowed^22^ nor group walks, shopping^23^ and various other activities. For these reasons, the level of outdoor noise significantly decreased and moved inside people’s houses^24,25^.

Noise can contribute to people’s stress levels. It is known that noise sensitivity can cause anxiety and depression^26,27^. However, while these studies are based on high noise levels impacting individuals, during the COVID-19 lockdowns, the noise level decreased significantly for a negative reason due to the context. In such situations, low noise levels may produce disagreements, dislikes and confounding sensations, because (1) people are exposed to this situation for the first time in their lives, (2) this event is not caused by a pleasant event and may be connected to a threat, (3) they are forced to sense this noise reduction, and (4) they perceived this situation from their homes and not from parks, historical city centers or places where individuals are usually not bothered by urban noise.

On the other hand, individuals may prefer the new soundscape because they can enjoy quieter outdoor (and thus indoor) conditions. Lower outdoor sound levels provide more indoor silence, especially in isolated houses or small blocks of flat. Furthermore, nature sounds, which are typically more appreciated, never stopped during the lockdown^28^, but instead actually increased^29^.

To the authors’ knowledge, at present many papers deals the measurement of the urban noise decreasing, pointing out how anthropic activities do influence this parameter. On the other hand no research focused on asking people if they realize and sensed that something was really changed in urban noise condition and whether they like or not this new and unexpected situation. People’s opinion is really important because if individuals changes habits and routine then it would be possible to obtain also better outdoor and indoor environments. The global change affected the general public everywhere, giving the chance to scientists to study this new phenomenon and the ideas and feelings caused in people’s minds. Results could be used to modify our actual way of living, already knowing if the new situation would be appreciated and tolerated by citizens and urban users.

For these reasons, the aim of this work is to respond to the following scientific questions: did people realize and sense noise differences during the first covid-19 pandemic lockdown? If so, was this the case for both indoor and outdoor conditions? If they sensed a difference, was it appreciated? And if yes, did they want this noise condition to be maintained in the future?

## Methods

In order to understand if some differences in the urban noise were present during the pandemic, field measurements were carried out in a city located in the northern part of Italy, where the lockdown was first applied in Europe. The city is populated by at about 40,000 persons with an area of 37 km^2^, including a high speed train railway, highway road, historical city center and a two hospitals. This European region was one of the earliest where the COVID-19 pandemic was detected and a full lockdown was imposed from early march 2020 to early May 2020.

**Figure 1:**
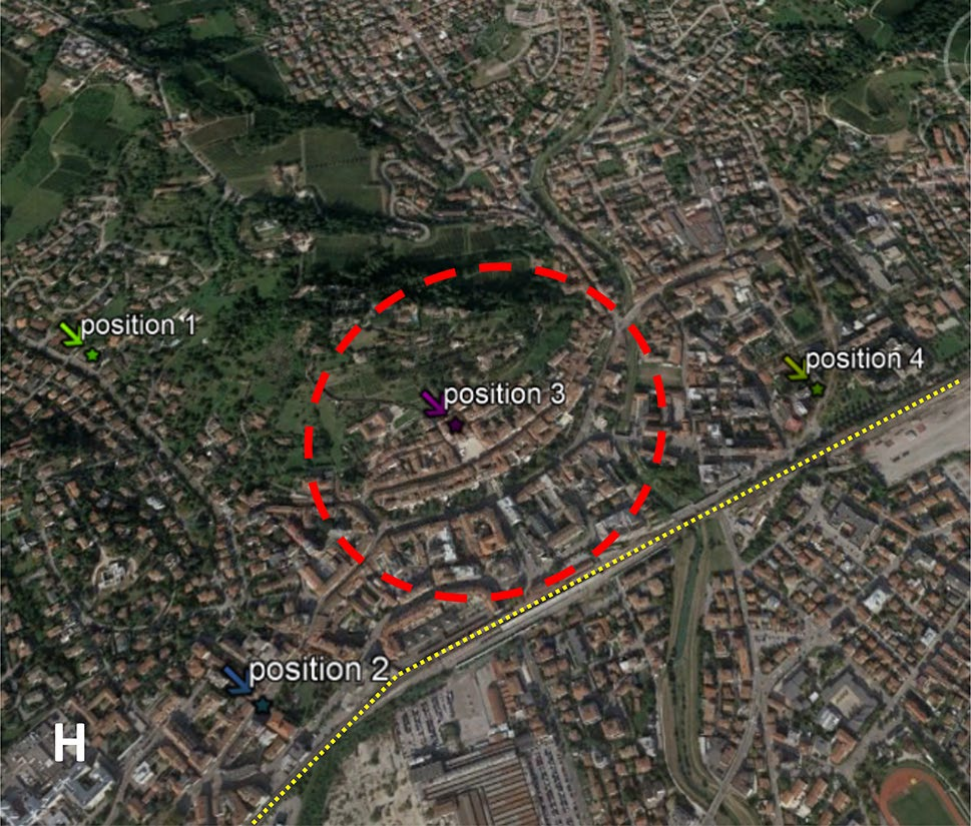
Italian city map. Dotted purple line identifies the historical city centre, while yellow one represent high-speed railway; capital H represents the Hospital position. Map realized using Google Earth version 7.3.3.7786 (32-bit), Maps data: Google, ©2021 CNES/Airbus, European Space imaging, Landsat, Copernicus, Maxar Technologies.

In this study, it is important to understand if really outdoor noise levels have been significantly decreased because of anthropic activities stop and traffic reduction. If this difference is demonstrated, we can be sure that the answers collected by survey reflect what was really sensed by respondents. To this aim, four measurement positions were chosen within the urban territory in order to simultaneously measure the day and night Sound Pressure Levels (SPL). In order to indicate results using one single index, L_den_ and L_night_ are utilized^30^. Test locations where chosen as to represent both the historical city center and the suburban areas (Fig. 1) and were located as follows:

- Positon 1: 20 m from the ground
- Position 2: 13 m from the ground
- Position 3: 14 m from the ground
- Position 4: 22 m from the ground.

**Figure 2:**
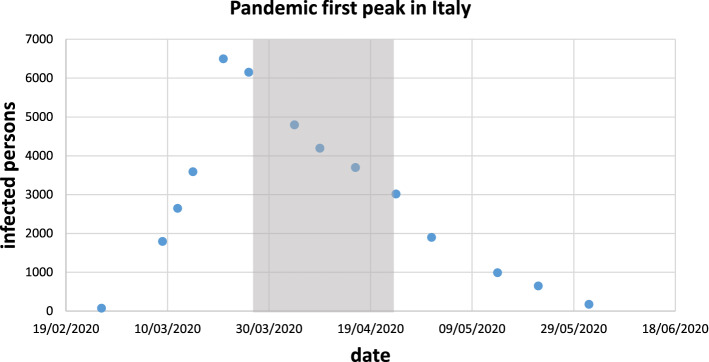
Peak verified in Italy during the first pandemic step. Dots: numbers of infected people per day. Light grey area: period of sound pressure level measurements.

Positions 2 and 4 where intentionally selected close to the railway line, in order to also assess this kind of noise. Position 2 is also close to the main city hospital, which was devoted to COVID-19 patients. Measurements started on March 17th and ended on April 15th, 2020. This time span was selected since the Italian Government forced schools to close on February 23rd, on the 9th March all traffic movements were banned except for urgencies, on the 11th most of the shops were closed and on the 22nd March 2020 complete lockdown was imposed. Within this period, the pandemic peak was reached as depicted in Fig. 2. Thus, results are representative of the increasing and decreasing infection rate.

No measurements before the pandemic are available in this area. Thus, results are compared to literature ones. In particular Guski et al. in their literature review^31^ reported many models and measurements of urban noise. It is evident that differences in urban environment are possible. Here, we can infer that anthropic and transportation noises are the most predominant active sources and for a city of at about 40,000 inhabitants a L_den_ range of 45 dB(A) − 80 dB(A) is expected.

In addition, in their study Stewart et al.^32^ proposed a method of background noise calculation usable in cities with a density range of 230 – 5000 persons/km^2^. The equation is reported below (Eq. (1)):

1 $${\text{L}}_{{{\text{dn}}}} = 17.9 + 10\log (\uprho )\quad \left( {{\text{dB}}\left( {\text{A}} \right)} \right)$$

This model was also confirmed by Gaja et al.^33^ in their extended work realized in Spain. In order to convert L_dn_ in L_den_ and L_night_, the conversion proposed by Brink et al.^30^ are applied in the calculation of the parameters and compared to the measured ones. In order to compute values, actual inhabitant’s density was utilized, using the data referred to the square kilometer where the each measurement took place.

At the same time, an international survey was developed and sent all over the world. This was intended to investigate how people react to the new unexpected outdoor and indoor noise conditions and to analyze if this was a disturbing or a pleasant situation.

The proposed questions deal with the assessment of people’s feelings in relation with urban and indoor noise modification, including ratings of the perceived amount of the variation (Q1-Q5), evaluation of their own sensations (Q6-Q7), the current preference (Q8-Q9) and future wishes (Q10-Q11). Four more questions were added at the end, asking to specify age, gender and home typology and location. The complete questionnaire is reported in Table 1.

**Table 1:** Proposed survey.

ID	Question
Q1	In this period of COVID-19 emergency lock down, have you sensed any modifications level, related to NOISE?
Q2	Do you agree with the following statement: URBAN noise now has not changed compared to what you could hear before the COVID-19 emergency lock down
Q3	If you sensed some difference in the URBAN noise level during this COVID-19 emergency lock down, can you please RATE this change?
Q4	Do you agree with the following statement: the INDOOR noise in your home environment now has not changed compared to what you could feel before the COVID-19 emergency lock down?
Q5	If you sensed some difference in the INDOOR noise level your home environment during this COVID-19 emergency lock down, can you please RATE this change?
Q6	Could you rate the sensation you perceive due to URBAN noise present in your city during the COVID-19 emergency lock down in comparison to what you were used to perceiving before the COVID-19 emergency lock down? Please use the graduate scale, where 1 is "great concern" and 5 is "well-being"
Q7	Could you rate the sensation you perceive due to INDOOR noise present at your home during the COVID-19 emergency lock down in comparison to what you were used to perceiving before the COVID-19 emergency lock down? Please use the graduate scale, where 1 is "great concern" and 5 is "well-being"
Q8	If you could change your home during the lock down COVID-19 emergency because of URBAN noise, you would prefer
Q9	If you could change your home during the lock down COVID-19 emergency, because of INDOOR noise, you would prefer
Q10	Would you prefer the present URBAN noise condition to be maintained in the future?
Q11	Would you prefer the present INDOOR noise condition in your home to be maintained in the future?
Q12	Gender
Q13	Age
Q14	Home typology
Q15	Home location

All possible choices are listed in Table 2. It can be seen that questions could present different scales. Generally speaking, a 5-point Likert’s scale is preferred when evaluating questions related to respondents’ attitudes^34^. Thus, it was used for questions ID 2, 4, 6, 7, 10 and 11. For preference-related questions, a 4-point Likert’s scale was used, eliminating the “neutral” choice so as to force respondents to choose between available answers^35^. This was used in questions ID 3, 5, 8 and 9. For the remaining topics, the number of choices depended on the available possibilities.

**Table 2:** Survey possible choices per question.

Index	Question	First response	Second response	Third response	Fourth response	Fifth response
General topic	Q1	Yes, related only to urban noise	Yes, related only to indoor noise at my home	Yes, both related to urban and indoor noise	No	–
Noise variation	Q2	Strongly disagree	Disagree	Neutral	Agree	Strongly agree
	Q3	Much noisier	Noisier	–	Quieter	Much quieter
	Q4	Strongly disagree	Disagree	Neutral	Agree	Strongly agree
	Q5	Much noisier	Noisier	–	Quieter	Much quieter
Sensation	Q6	1 (great concern)	2	3	4	5 (well-being)
	Q7	1 (great concern)	2	3	4	5 (well-being)
Living place variation	Q8	A much quieter place	A quieter place	–	No change	A noisier place
	Q9	A much quieter place	A quieter place	–	No change	A noisier place
Preference	Q10	Definitely not	Possibly not	Neutral	Possibly yes	Definitely yes
	Q11	Definitely not	Possibly not	Neutral	Possibly yes	Definitely yes
Gender	Q12	Male	Female	–	–	–
Age	Q13	19 or less	20–39	40–59	60–79	80 or more
Home typology	Q14	Detached house	Townhouse/terraced house	Apartment building with up to 10 apartments	Apartment building with up to 30 apartments	Apartment building with more than 30 apartments
Home Localization	Q15	City centre	Suburbs	Countryside/seaside/mountains	–	–

Results were examined by means of both accumulated percentage and statistical analysis. Italian and international answers collected were first checked to understand if there could be criticalities and inconsistencies. A non-parametric Mann–Whitney approach was chosen in order to manage unknown data distributions related to independent samples^36^, considering significance levels of both 1% and 5%. In this view, firstly a control sample with geographically limited respondents was selected. The limitation was applied only to the answers given by people living near the places where sound pressure measurements were made. In this light, we were able to connect their reactions to the measured noise level. A Mann–Whitney analysis was then performed to study if there were significant differences between this control sample and the rest of Italy. If no noteworthy variance is found, we can conclude that results can be generalized to the whole of Italy. Following this, another Mann–Whitney test was performed between overall Italian and International surveys, in order to extend results to a global context. Furthermore, CART Decision Trees^37^ were used to identify correlations between answers, dividing the data recursively according to the variable that produced the greatest increase in homogeneity in the results after partitioning. This procedure was implemented according to four steps:

1. the questions related to urban topics (Q2, Q3, Q6, Q8 and Q10) were treated as an endogenous variable, while questions from Q12 to Q15 were considered independent;
2. the questions related to indoor topics (Q4, Q5, Q7, Q9, Q11) were treated as an endogenous variable, while questions from Q12 to Q15 were considered independent;
3. the general initial question Q1 was considered endogenous and the urban questions were considered independent;
4. the general initial question Q1 was considered endogenous and the indoor questions were considered independent.

CART Decision Trees is used as predictive model approach. A decision tree is useful from observations point of view about an item (represented in the lines or connections) to conclusions about the item's target value (represented in the rectangles or circles—tree “leaves”). Tree representations can manage a discrete dataset and are also named “classification trees”; in their structures, “leaves” are used to embody class labels and connection embody conjunctions of topics linking those labels. Decision trees used very often because of their clear representation of the treated topics^38^. The software “wessa.net” was used to plot results^39^.

Both for Mann-Witney tests and for CART regression trees, numbers from 1 to five were associated to responses, choosing 3 as neutral answer. When a 4-points Likert’s scale was used, the 3 score was not considered.

### Ethical approval

This research was conducted and concluded with the approval of the Free University of Bozen Ethics Committee which did not identify any ethical issues and thus considered the research as ethical. The study procedure was designed in compliance with the Declaration of Helsinki. Informed consent was attained from all participants of the survey and a clear aim and scope of the questionnaires was included in the first page of the on line platform. All participations were volunteer and no obligation was made to start, fill in and conclude any of the survey.

## Results

### Sound pressure measurements

**Figure 3:**
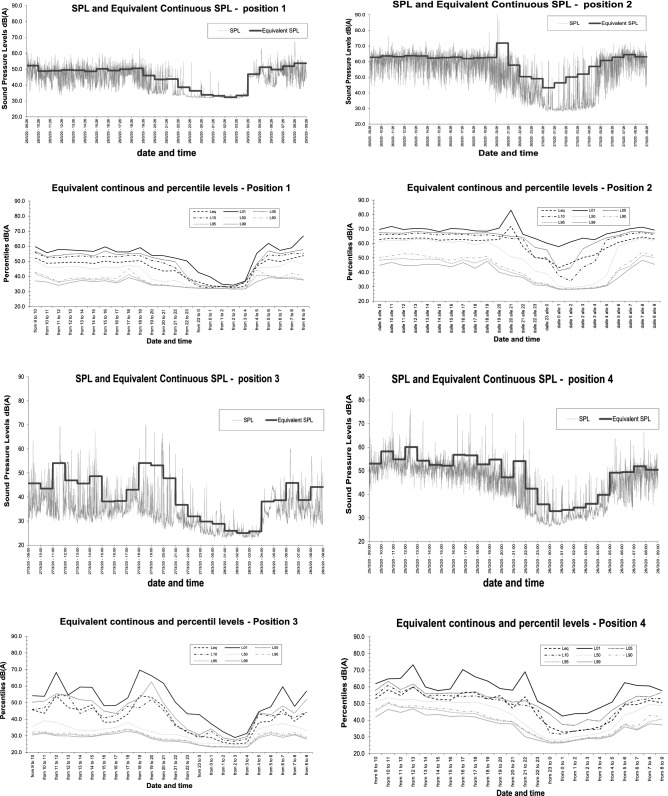
Sound pressure level measurements results.

Sound pressure levels measured on the above-mentioned positions are reported in Fig. 3, where, for the sake of brevity, only a representative working day for the four positions is depicted. All working days presented almost the same trends and values. For every measurement point, instantaneous sound pressure level and hourly equivalent sound pressure level are represented for a 24 h period. In addition, hourly percentile levels are displayed and compared to equivalent continuous SPL. These are very useful to understand how noise is moving and modifying during the whole day and night^40^.

Values of L_den_ and L_night_ are reported in Table 3 and compared to the ones calculated using Eq. (1).

**Table 3:** Overall results for the four measurements positions (sound pressure levels in dB (A)).

Position number	L_den, meas_	L_den,calc_	L_night,meas_	L_night,calc_
1	50.8	53.9	45.6	45.9
2	67.2	57.9	57.0	49.9
3	43.3	52.7	38.4	44.7
4	49.1	55.7	43.9	47.7

### Online surveys

**Figure 4:**
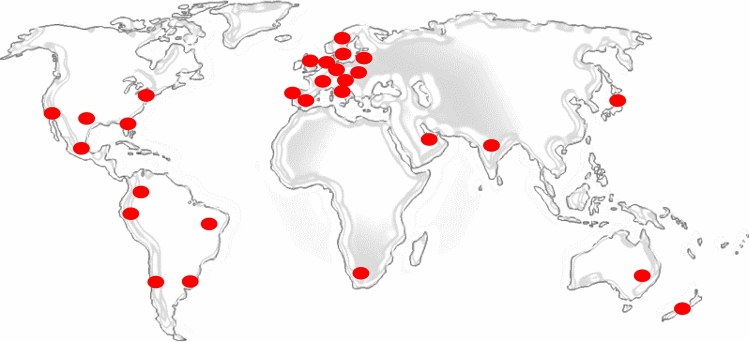
Respondents’ geographical distribution. Red spots roughly identify state of provenance (map^41^ freely modified using Microsoft Word).

Respondents’ geographical origin are shown in Fig. 4. Here, it is possible to see the international distribution of the collected answers. Red spot represent the capital city where the country belongs.

**Figure 5:**
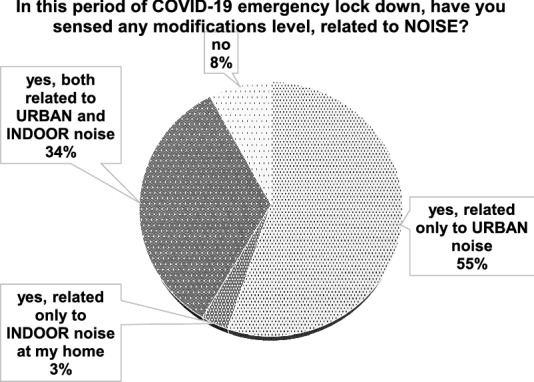
Italian results: general topic.

Overall, more than one thousand independent responses were collected. The control sample consisted of 144 answers. Percentage results for Q1 Italian responses are depicted in Fig. 5, where it possible to verify trends related to the general topic.

**Figure 6:**
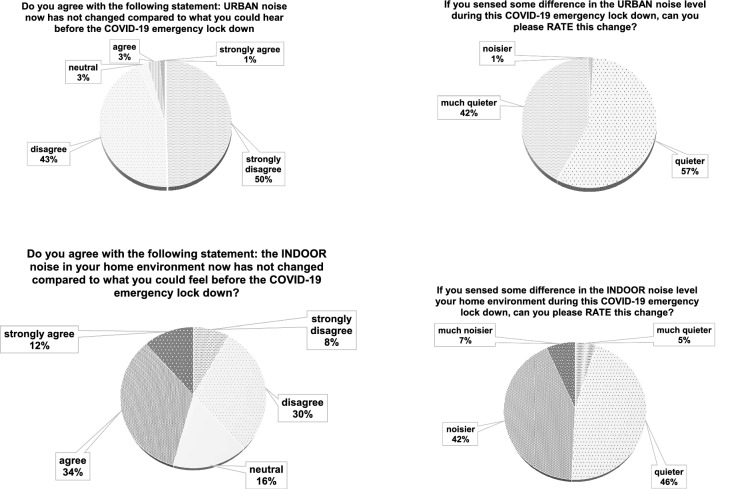
Italian results: comparison between indoor and outdoor noise variation perception.

In Fig. 6, answers related to Italian respondents inherent to noise variation (Q2, Q3, Q4 and Q5) both in indoor and outdoor environments are depicted.

**Figure 7:**
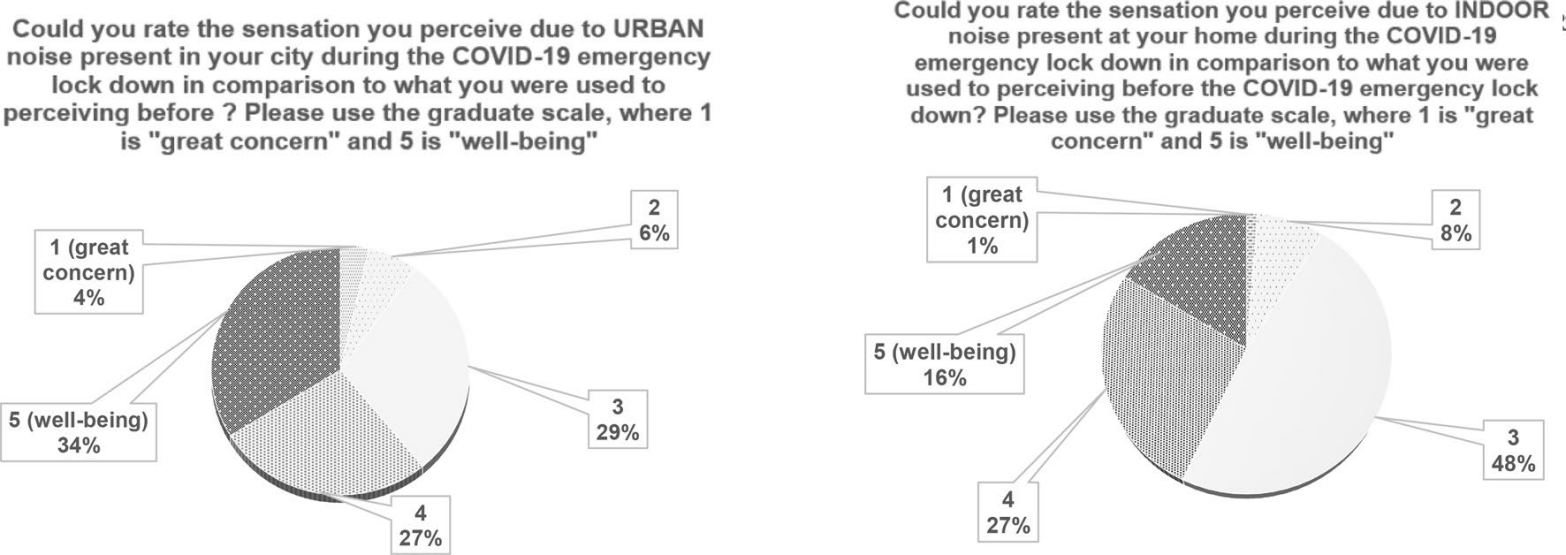
Italian results: comparison between outdoor and indoor noise variation sensation.

When talking about sensation, Q6 and Q7 Italian responses are depicted in Fig. 7. Here, the comparison between urban and indoor environment is presented, in relation to perceived sensation caused by noise variation.

**Figure 8:**
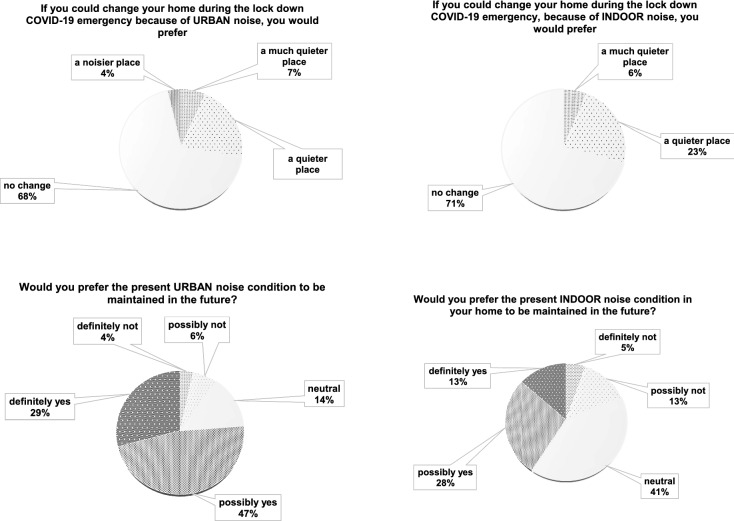
Italian results: comparison between outdoor and indoor noise variation future intention.

Questions 8 and 9 (Q8 and Q9) investigate the preference of people concerning a possible home change, because of urban noise or indoor noise variation. The last two questions (Q10 and Q11) ask individuals about the chance to maintain this noise situation in the future, regardless of the pandemic. In Fig. 8, Italian results are depicted.

**Figure 9:**
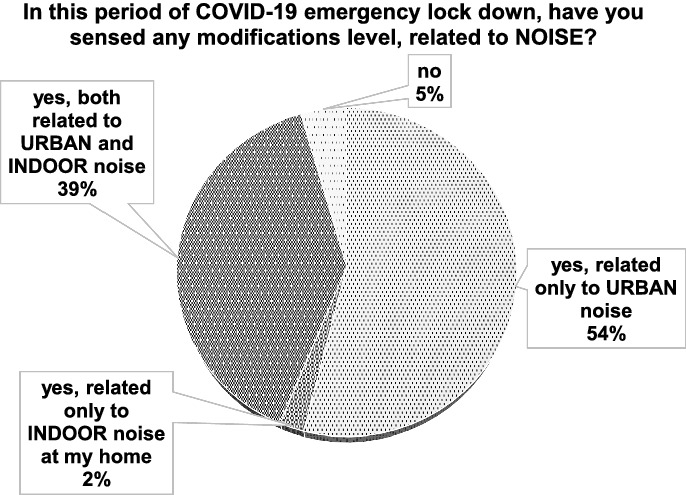
International results: general topic.

From Fig. 9, International results are listed. Here, the general topic responses are highlighted.

**Figure 10:**
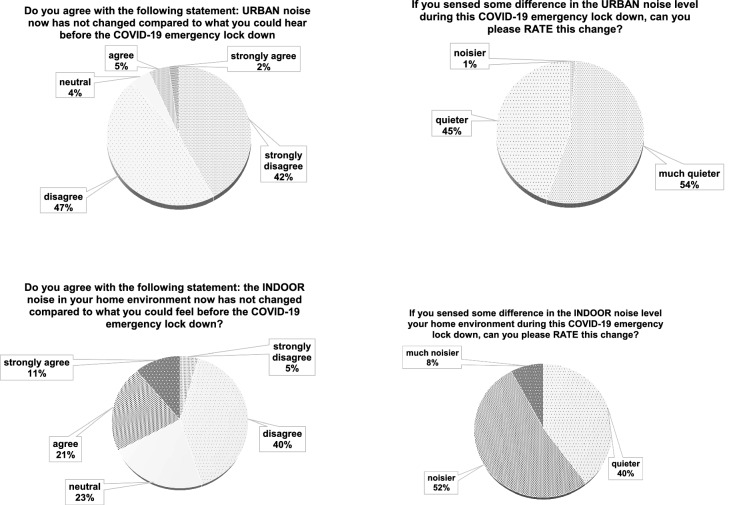
International results: comparison between outdoor and indoor noise variation perception.

In Fig. 10, Q2, Q3, Q4 and Q5 international results are reported, concerning the change of indoor and outdoor noise caused by the lockdown.

**Figure 11:**
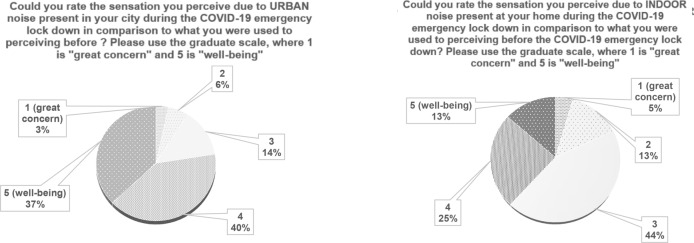
International results: comparison between urban and home variation sensation.

The sensation caused by pandemic on urban and indoor noise is investigated by means of Q6 and Q7. In Fig. 11, international responses are depicted.

**Figure 12:**
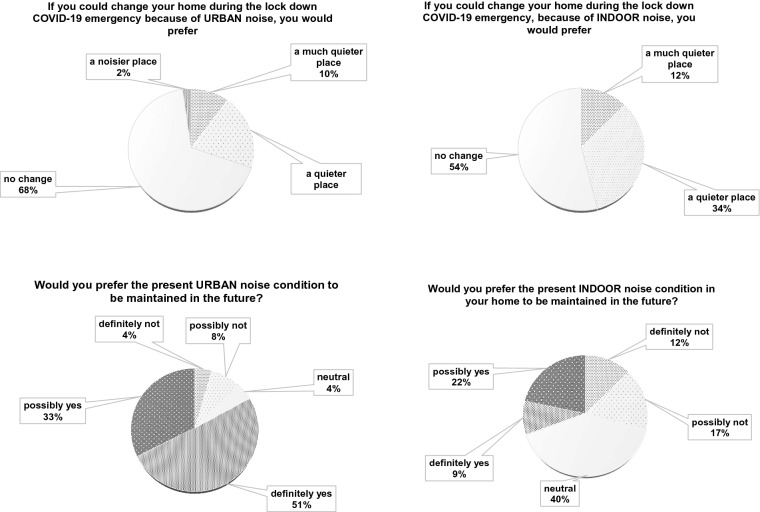
International results: comparison between urban and home variation future preferences.

Home change is proposed in Q8 and Q9 and in Fig. 12 related international outcomes are highlighted. Lastly, in Q10 and Q11 individuals are asked to evaluate if they would maintain the noise situation in the future.

### Statistical analysis

Results from the Mann–Whitney test are reported in Table 4 for the comparison between the control sample and the overall Italian responses. In Table 5, the evaluation between overall Italian and International responses is listed.

**Table 4:** Mann–Whitney test. Comparison between Italian control group and overall responses.

Index	Question	U	z-Score	*p*	Significant at *p* < 0.01	Significant at *p* < 0.05
General topic	Q1	49,788.00	− 0.145263	0.884503	No	No
Noise variation	Q2	50,079.00	− 0.039590	0.968420	No	No
	Q3	49,046.00	− 0.471138	0.637542	No	No
	Q4	49,572.00	− 0.219695	0.826108	No	No
	Q5	42,525.00	− 3.145930	0.001655	Yes	Yes
Sensation	Q6	46,471.50	− 1.433485	0.151719	No	No
	Q7	46,788.50	− 1.345639	0.178419	No	No
Living place variation	Q8	53,297.50	1.471246	0.141225	No	No
	Q9	54,192.00	1.942874	0.052031	No	No
Preference	Q10	51,239.00	0.449852	0.652817	No	No
	Q11	48,106.50	− 0.797788	0.424993	No	No

**Table 5:** Mann–Whitney test. Comparison between Italian and International responses.

Index	Question	U	z-score	*p*	Significant at *p* < 0.01	Significant at *p* < 0.05
General topic	Q1	29,601.00	− 1.016484	0.309399	No	No
Noise variation	Q2	29,714.00	− 1.821086	0.068593	No	No
	Q3	32,326.00	0.516259	0.605673	No	No
	Q4	29,808.00	− 0.822288	0.410913	No	No
	Q5	35,077.50	1.972825	0.048515	No	Yes
Sensation	Q6	27,720.00	− 1.891932	0.058500	No	No
	Q7	249,218.00	− 0.000070	0.999944	No	No
Living place variation	Q8	29,974.50	− 0.860690	0.389409	No	No
	Q9	25,856.00	− 3.352954	0.000799	Yes	Yes
Preference	Q10	24,881.00	− 3.429028	0.00060	Yes	Yes
	Q11	35,354.50	2.024262	0.042943	No	Yes

**Figure 13:**
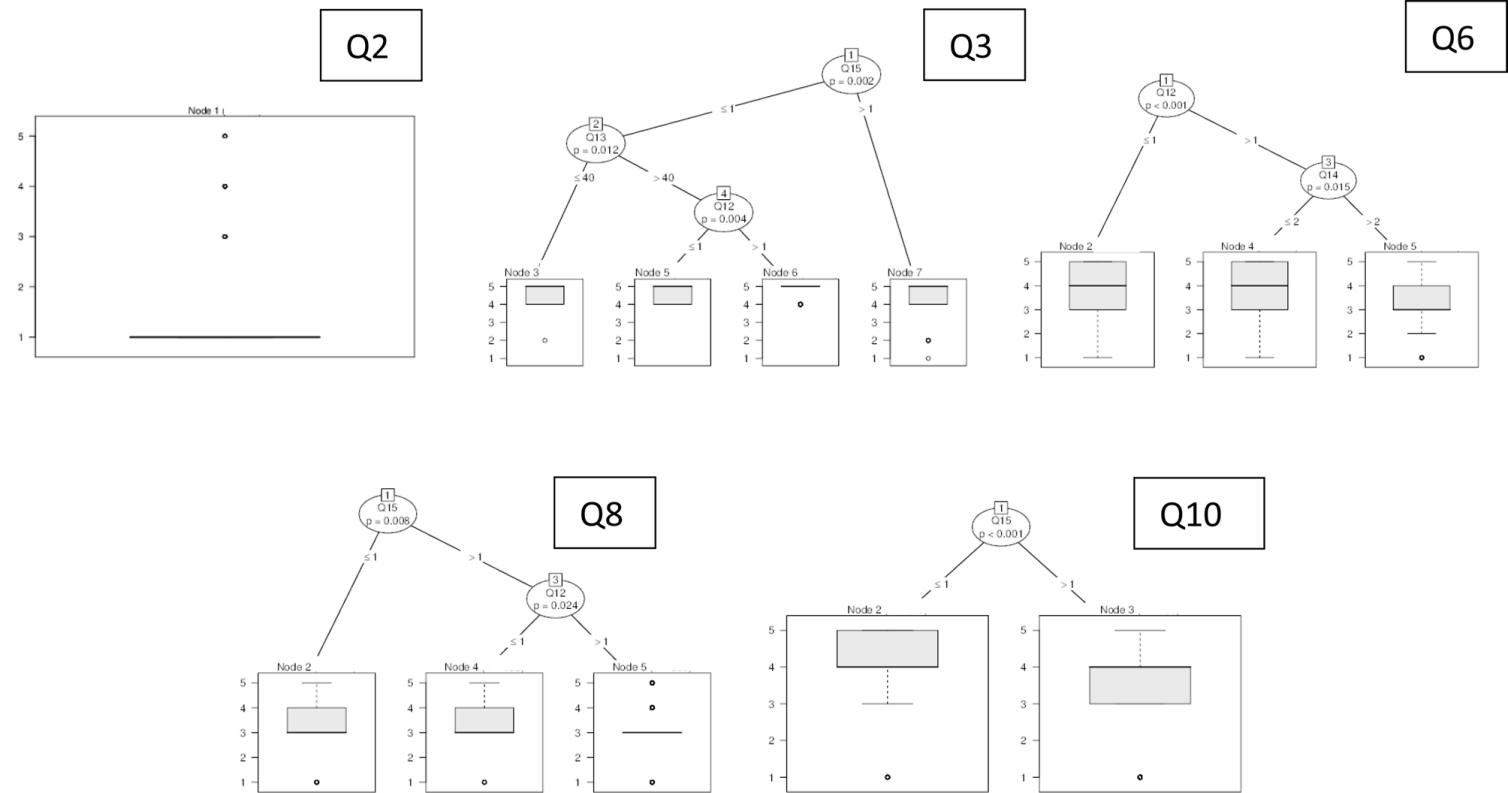
Decision regression trees for urban environment questions.

**Figure 14:**
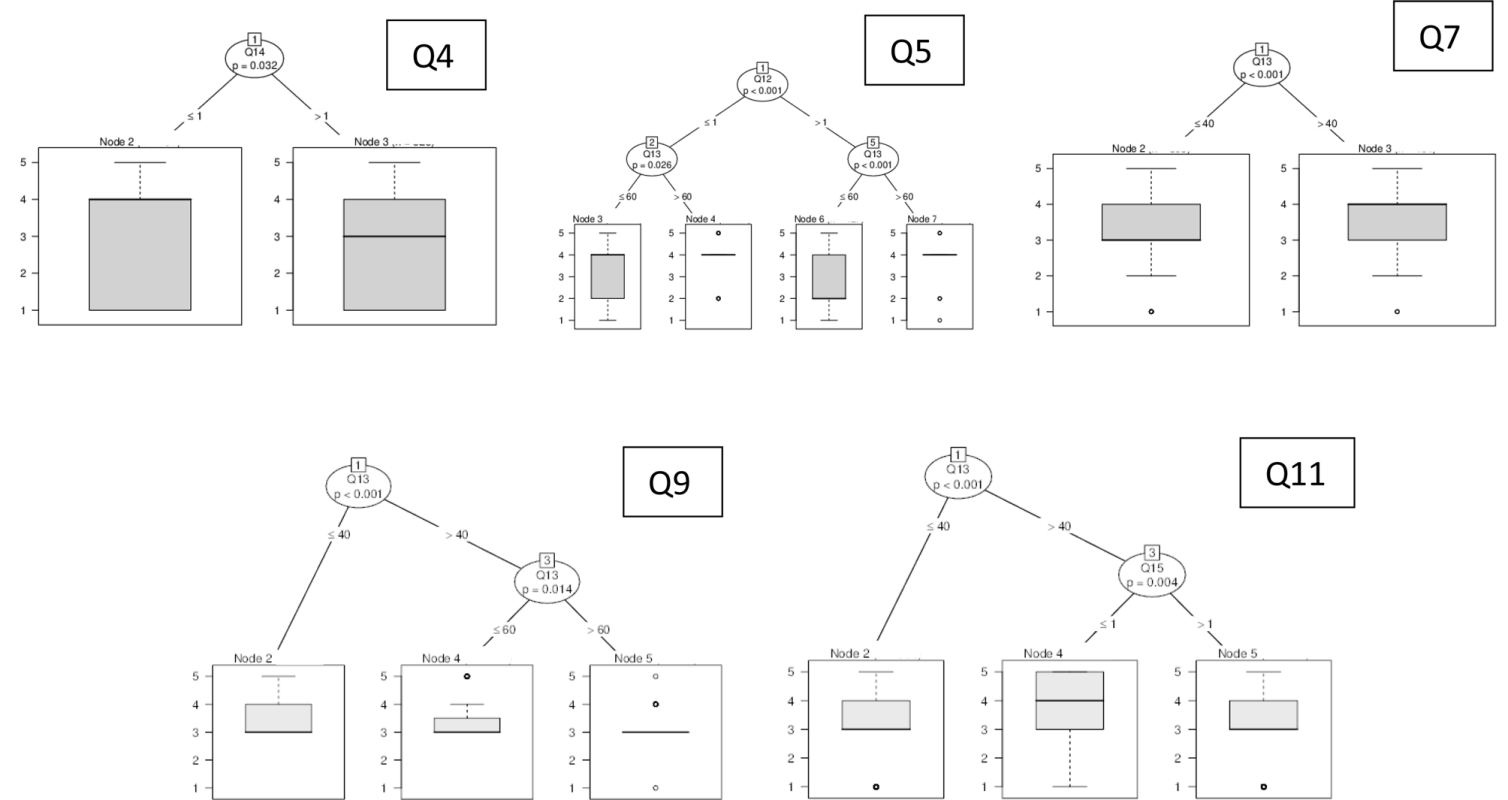
Decision regression trees for indoor environment questions.

The decision regression trees based on the questions focusing on urban environment (Q2, Q3, Q6, Q8 and Q10) are reported in Fig. 13 (step 1), while the indoor environment questions are depicted in Fig. 14 (step 2).

**Figure 15:**
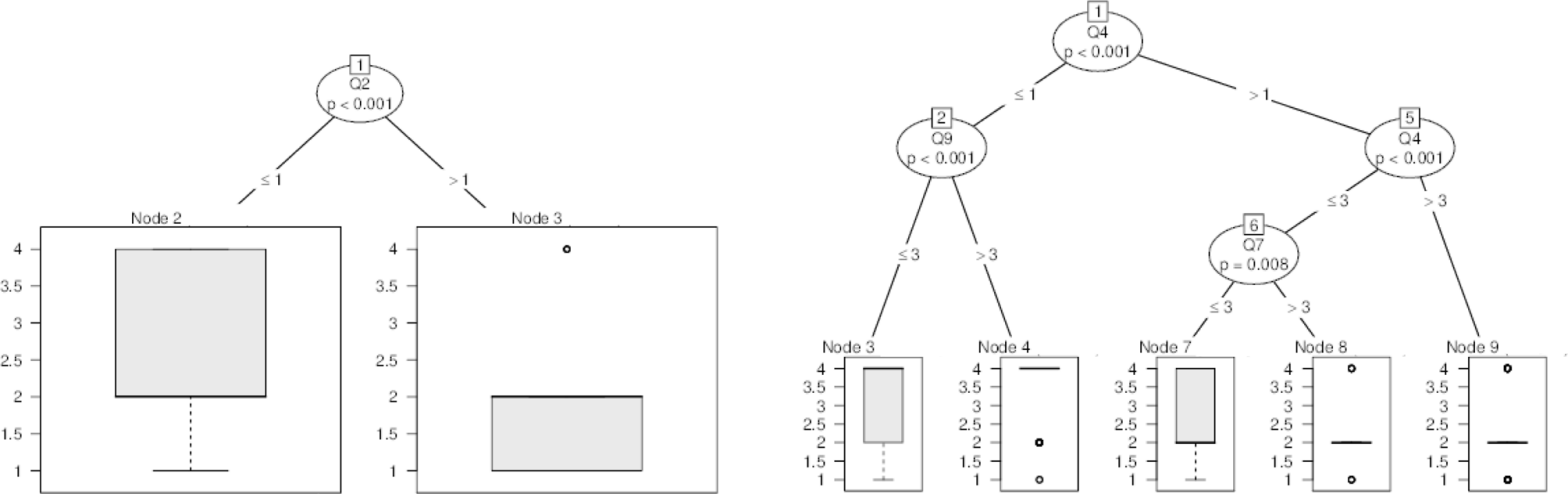
Decision regression trees for urban and indoor environment questions related to Q1.

The decision regression trees for Q1 comparing urban environment questions (Q4, Q5, Q7, Q9 and Q11) (step 3) and indoor environment questions (step 4) are depicted in Fig. 15.

## Discussion

Respondents participated from North America (the USA and Mexico), South America (Brazil, Ecuador, Peru, Colombia, Chile and Argentina), Europe (Italy, Austria, Spain, Portugal, France, the UK, the Czech Republic, Sweden, Latvia, Lithuania, Switzerland, Belgium, Germany, The Netherlands, Bulgaria), Asia (Dubai, India and Japan), Africa (South Africa) and Oceania (Australia and New Zealand). This shows a very wide participation in the survey and implies that, as has been demonstrated several times^42^, the pandemic affected most countries in the world. This also evidences how for the first time in recent history most people faced similar and highly impacting environmental variations at same time, which they could evaluate and grade.

### Sound pressure measurements

The European Commission states two main indicators in order to classify noise pollution: L_den_ and L_night_. The first indicator is used to represent, with a single number, the sound pressure level measured during the whole day, while the second denotes the level verified only during the night (23.00–7.00). For the first one, a reference threshold of 55 dB(A) is set, while 50 dB(A) for the second^43^. When this limit is surpassed, it is considered to be noise pollution. As evidenced in Table 3, in almost all the positions these limits are fulfilled. Only in position 2, a very high sound pressure level assessed. This is due to the fact that the hospital is very close to this position, and the street where the sound level meter was positioned was the quickest way to reach the hospital. The higher number of ambulances at any time of the day in that period and their proximity to the microphone influenced the measurements.

Interestingly, the lower values were found in position number 3. Here, only traffic-free roads are present and no nature (very few trees, no meadows, very few and small gardens, no flowerbeds). In Fig. 3, it is evident how from 20.00 in the evening, the sound pressure level started to decrease reaching hourly values of 25.0 dB(A) from 2.00 to 3.00 in the morning. This significantly low value is usually measured during the night, within a well-insulated dwelling not surrounded by noteworthy noise sources^44^. This is quite interesting since measuring such a low sound pressure level in outdoor conditions shows how it is possible to have a very quiet outdoor soundscape when anthropic noise has almost completely stopped and where no contribution from natural sound is expected.

On the other measurement positions, closer to streets where some traffic and nature sounds were present during the day and night, measured values show higher sound pressure levels, but still significantly lower than normal values. Percentile trends, as well as hourly and instant values, show how similar events happened in all positions (except for positions 2 for the above-mentioned reasons), but with lower levels where no traffic is present. This fact evidences that in the whole city the same outdoor noise was present and could be sensed and perceived.

In general these levels are significantly lower than the usual ones characterizing urban areas all over the world^45–48^. Moreover, these data confirm that the higher noise levels in normal conditions depend on human activities.

### Survey general results

It is evident how the question Q1 related to the general topic shows the same trend for Italian (Fig. 5) and International results (Fig. 9). This means that the outdoor sound field variation level was sensed everywhere and was not limited to single areas or countries. It is interesting here to note that also the indoor acoustic situation also seems to have changed for one third of the respondents. This may be due to the fact that they were made to stay at home during the whole day. Thus, the noise from neighbours as well as the usual noise was perceived in contrast with the period before the first pandemic wave, when many respondents were at work during the daytime. Furthermore, since everybody was at home, indoor noise was also likely to be higher than the one perceived without a lockdown or with only partial restrictions.

For Q2, similar result trends can be seen when comparing Italian (Fig. 6) to International responses (Fig. 10). For Q3, both surveys highlight that almost all respondents perceived the urban environment as quieter and that the international results show a higher occurrence of “much quieter” compared to the Italian survey responses. In Q4 about the indoor environment, Italian outcomes show that about half of the respondents agreed about a change in perceptions, while only one third of the international ones agreed with the question statement. On the other hand, about one half of the International responses disagreed with the statement. This means that the two surveys do not show a complete accordance on this topic. This is confirmed by Q5 with the Italian results showing half of the people sensing their home to be “quieter”, while the other half “noisier”. The international responses highlight that most of the people perceived their homes as noisier.

From the point of view of the feelings caused by the urban and new indoor noise conditions, answers to Q6 and Q7 show similar trends (Figs. 6, 11), evidencing how people liked the quiet situation caused by the lockdown situation. When asked about possible variations of home locations as in Q8, Italian results clearly lean towards a negative answer (Fig. 7). In the International responses (Fig. 12) half of the respondents would prefer a “quieter” or “much quieter place”. This fact probably emphasizes how in Italy, during the first pandemic wave, the lockdown was stricter than in other countries as found in literature^13^ and thus impacted more significantly on outdoor noise levels.

As an overall finding, it can be affirmed that anthropic noise is definitely perceived by almost all respondents as a cause of annoyance and stress. Very few people consider a quieter or much quieter situation to be an alarming or concerning sensation, even if it caused by a pandemic and thus a negative condition. In this light, even if living in a very hazardous moment, full of stressful news and circumstances, people relate silence or at least reduced anthropic noise to a pleasant perception. When asked if they wanted to maintain this level of urban noise in the future, only 10% of the respondents opposed this, while around 90% of the answers were in agreement with the statement or at least neutral. This clearly means that people do appreciate a quieter urban environment and maybe for the first time they had the opportunity to perceive it close to their home, within their familiar environment and not just related to holidays or traditionally quieter places, like mountains, deserts or in general remote locations.

### Survey statistical analysis

Firstly, the analysis focused on the comparison between a smaller control sample and the overall Italian results. This sample considered 144 respondents, living in one of the very first complete lockdown areas in Italy. The survey was sent during April 2020 and results were received within the following 20 days. This time range was intentionally kept as short as possible in order to collect people’s feelings on what was still happening and not based on memories. After this, quickly, several countries (almost all in Europe) imposed total or semi total lockdowns, thus recreating what was presumably very similar outdoor and indoor situations in terms of noise levels.

For these reasons, the comparison between the control group and the overall Italian responses and, following this, the comparison between these Italian responses to the International ones can show if sensations confirmed by the objective measurements can be generalized to other geographical areas and countries. As discussed in the previous section, it is evident how all respondents sensed a significant difference in the outdoor environment. This is also confirmed by Fig. 3 and Table 3, where objective measurements are reported. We can therefore conclude that the control sample, which is also monitored by the field measurements, sensed a huge variation between current indoor and outdoor noise levels and pre-lockdown levels. Furthermore, we can also highlight from Table 3 that the control sample and Italian sample are in agreement most of the time, since no significant statistical variation appears for the relevant questions (from Q1 to Q11), with the exception of Q5. When analysing the possible statistical difference between Italian and International responses, in Table 4 it is possible to notice that no significant variation at any significant threshold is assessed in questions Q1 to Q11 for a significance related to p < 0.01. Considering a significance of p < 0.05, again Q5 shows a difference in the two samples. We can thus conclude that all countries experienced similar conditions and that sensed objective SPL outdoor decreasing, in comparison to the pre-lockdown situation.

The decision regression trees help understand what impact living habits, preferences and feelings had on respondents’ decision making process when answering the questions. This statistical regression is meant to be used when several variables are influencing an endogenous variable. Using regression, we are able to infer if some of the personal conditions could influence individuals’ responses. Nodes represent diverse decisions. In Fig. 13, the aim is to verify if there is some dependency on gender, age, home typology and home localization in answers to the questions about urban noise. It is evident that for Q2 the median is clearly focused on the “disagree” side with some outliers on the other answers. No influence of other parameters is found and nearly everybody thought that urban noise had changed, as depicted in Figs. 6 and 10. When moving on to Q3, things change and respondents can be sorted by home location. If they live in the city centre (≤ 1), they can be divided into younger people (≤ 40) or older people (> 40). In the first case their responses are focused on a boxplot comprised between “quieter” and “much quieter” answers, with the median on “much quieter”. Some outliers are present. When moving onto older respondents, a gender selection can be made. Males (≤ 1) are again focused on the “much quieter” and “quieter” answers with no outliers, while women (> 1) are more determined on “much quieter”. When people live in the suburbs or countryside/seaside/mountains they are not influenced by age or gender and the median is clearly fixed on “much quieter” with some outliers.

When investigating the perceived sensation related to urban noise (Q6), we can understand that males and females may have diverse opinions. Answers of male respondents are distributed in the whole range, even if the median is 4 (close to “well-being”). The responses from females can be divided according to home typology. If they live in a detached house or in a townhouse/terraced house (≤ 2) they completely agree with the males’ responses. However, if they live in more crowded buildings, the median moves to 3 and the boxplot also moves one step down. This means that perceived sensations of the urban noise could depend on home typology for female respondents.

Q8 deals with the individual’s preference for their own home and their willingness to change is due to the lockdown situation. In terms of urban noise, it can be seen that the home location plays the most important role, as expected. Accordingly, if one lives in the city centre, then the results fall within the 3 (no change)—5 (much noisier place) score with (the median is 3). When moving from city centres, a distinction between genders is possible. Consequently, males present the same situation as before, while women’s responses are clearly focused on “no change” (median = 3) with some outliers.

Respondents are further asked about their preferences in Q10, which refers to the chance to maintain the urban noise situation caused by the lockdown in the future. It is evident here how the only influencing parameter is the home typology. Even if the two presented cases include the same score ranges and the same median, it is evident how the boxplots are different. When living in the city centre people strongly prefer to maintain the situation in the future, while those living in the suburbs or in countryside/seaside/mountains do not stress this point.

Questions related to indoor noise were analysed in Fig. 14. Q4 is related to the noise variation within indoor environments. Responses are clearly influenced by home typology. Accordingly, even if the range is always spread over all scores, people living in a detached house show a median related to “agree”, while those who live in other types of houses show a more neutral response. This means that individuals living in homes that are more crowded sometimes sensed an increase in the indoor noise levels. Conversely, people living in detached houses did not perceive this difference. When rating this change (Q5), no influence of home typology or location is verified. Accordingly, only gender and age are relevant. Males and female responses vary when considering people under or over the age of 60. In the first case, the scores range is extended on all responses, the median is 4 (agree) and the boxplot is from “noisier” to “quieter”. Considering males over 60, the median is 4 (quieter) with only some outliers. The same results can be seen for females, except for the fact that the median of females under 60 is 2 (noisier). This means that even though both males and females under 60 present the same box plot and ranges, but rate the variation of the indoor noise differently, this difference disappears for those over the age of 60.

When studying the perceived sensation related to indoor noise (Q7), one can understand that only age is the influencing parameter. Accordingly, even if both cases present the same distribution in answers range, individuals under 40 Answers show a median equal to 3 while over 40 the median rises to 4 (closer to “well-being).

When asked if they would prefer to change their home during the COVID-19 lockdown because of indoor noise (Q9), individuals did not seem to be influenced by home typology or location but rather by age. Consequently, it can be highlighted that the median is always 3 (no change). Younger respondents (≤ 40) also preferred noisier places, while middle-age individuals (between 40 and 60) showed less preference and for the over 60 “no change” is strongly selected with some outliers. We can learn from these results that the younger the respondent is, the more a variation (noisier place) of the home is preferred.

Q11 investigates respondents’ preference for maintaining the indoor noise conditions in the future. The first node is represented by the age. For younger people (≤ 40) the median is focused on “no change”, featuring a boxplot comprising 4 (possibly yes) and a range extending from 3 (neutral) to 5 (definitely yes). When considering older people (> 40), the home location is relevant. People living in the city centre clearly prefer (median = 4) maintain in future the indoor noise perceived during the lockdown. Respondents living in suburbs or countryside/seaside/mountains show a median around 3 (neutral). These findings demonstrate how the urban noise influences the indoor noise levels. Accordingly, people living in the city centre are more prone to prefer the indoor noise caused by lockdown according to the Q10 results. Since it is acknowledged that city centres provide a noisier environment^49–51^ with respect to the suburbs or countryside/seaside/mountains, this parameter is demonstrated to be the one most influencing Q11 responses.

In the last two steps, the first general question (Q1) is considered as endogenous and dependent on the “urban” or “indoor” topics. In the first case, the only relevant node is represented by Q2. Accordingly, even if the median is always 2 (yes, related only to indoor noise at my home), two different scenarios appear. On the one hand, respondents choosing “strongly disagree” in Q2 never selected in Q1 “yes, related only to urban noise”. On the other hand, people choosing other options in Q2, only choose “yes, related only to urban noise” or “yes, related only to indoor noise at my home” in Q1. These results highlight that those who strongly disagreed with the fact that the urban noise had not varied during the lockdown, also perceived that the indoor noise varied. This could be due to the fact that many neighbours or family components were forced to be at home during the lockdown, thus increasing indoor noise.

In the second case (fourth step), the first relevant node is represented by Q4. Here, as for the third step presented above, there are differences related to responses associated to “strongly disagree”, with respect to the other options. Another node is represented by Q9. Accordingly, respondents could be divided into two groups: individuals who would choose a quieter place and others that prefer a noisier home. In both cases the median is 4 (“no changes”), but in the first case, the range comprises all scores and the boxplot is comprised between 2 to 4, while in the second case only the 4 value is identified with some outliers. On the other hand, again results from Q4 can be divided into two groups: under 3 or over 3 (“neutral”). The first ones provide another node represented by Q7, which offer a median of 2 (“yes, related only to indoor noise at my home”) thus highlighting that those who sensed that the indoor noise was linked to “concern” also felt differences in urban noise. In contrast, those who had a positive feeling about noise variation, related it to the indoor noise.

### Responses to the research questions

In the introduction, we stated some research questions, which now can be precisely and clearly answered. “Did people realize and sense noise differences during the first covid-19 pandemic lockdown?”. It can be surely answer that yes, people do realize that noise was different during the pandemic. We can see that from questions Q1, Q2, Q3 and Q4 results respondents do express a difference in noise sensation. No significant difference was found between people living in different countries so we can sure determine that this condition was common in most areas.

“If so, was this the case for both indoor and outdoor conditions?”. Yes, both for indoor and outdoor conditions people expressed this difference in noise sensation. We were also able to define using Q5 results that most respondents felt the outdoor noise quieter, while for the indoor noise this condition was not confirmed, mostly because people blocked in their house produced noise felt by neighborhood.

Using the responses of Q6, Q7 and Q8 we can answer the question “If they sensed a difference, was it appreciated?”. People did like the new situation in spite of the hazard caused by the pandemic. Furthermore, they really also appreciated the calm outdoor environment when living in detached house, while they asked to move to quieter houses when living in flat blocks.

The last posed question is “did they want this noise condition to be maintained in the future?”. Almost all respondents declared that they would maintain the outdoor condition in the future, preferring to confirm the quieter situation in the 90% of the answers.

### Limitations and future developments

In this paper, the individuals’ preferences towards indoor and outdoor acoustic environment are investigated. Many new findings were listed and discussed. As every scientific work, some limitations have to be highlighted. The first limitation is that we do not have sound pressure measurement done where we wanted. Clearly during a worldwide lockdown we could not move from our homes. Anyway, using literature, an extension of these measured results was possible. Another limitation is related to the sample. Even if we had many answers, some more would helped to respond to other scientific questions, not included in this paper. A final limitation was that there was no chance to interview a population not exposed to the lockdown since it was a worldwide condition. However, we feel significantly confident that the statistical analyses show robust and reliable results.

Future developments will be related to the precise investigation of individuals’ sensations and preferences towards simulated and real soundscapes, in order to provide more data on outdoor and indoor comfort related to low sound pressure levels exposure.

## Conclusions

This work investigated the influence of the first COVID-19 pandemic wave on urban noise and its effect on people’s reactions. The research aimed to understand whether outdoor noise modifications were possibly sensed by individuals and how these modifications were perceived.

For these reasons, measurement results and survey were realized and results were collected and analysed by means of statistical analysis.

The main findings show that significant outdoor noise variations were found and that people clearly sensed noise variations both in urban and indoor conditions. Statistical analysis comparing local, national (Italian) and International (worldwide) responses undoubtedly showed no significant differences, except for one question. We can therefore determine that generally individuals perceived and rated the urban noise difference caused by the COVID 19 pandemic in a consistent way. Furthermore, we can also conclude that generally people liked the new quieter conditions, even if it was caused by a hazardous event and they also clearly wanted to maintain the outdoor noise levels in the future.

Descriptive and inferential statistics and decision regression trees present the following other interesting findings:

- Sound pressure level measurements revealed daytime and nighttime results, which are very different from what is usually found in cities. Here, an hourly outdoor level of 25 dB(A) was measured during the night, where usually a very different sound scape is present. This demonstrates how anthropic noise and traffic are what affect our cities and in general the urban noise. If we want to reduce it in ordinary life, significant measures have to be considered by policy makers and voluntarily actuated by citizens;
- almost 75% of respondents clearly identified a significant reduction in the outdoor noise level worldwide, in the same period;
- the outdoor variation also influenced the indoor noise for at least one third of the respondents; accordingly, they felt an increase in this parameter, presumably due to the fact that residential buildings were more crowded, because of the lockdown and of the reduced masking effect of the outdoor noise;
- no external parameters, such as age, gender, home location or typology influenced people’s urban noise perception: everybody clearly sensed everywhere that noise significantly decreased;
- when rating the change, as expected home location has an impact on answers, even if they mostly change from “quieter” to “much quieter” for people living in the suburbs, country side or even the mountains;
- when rating the perception, a gender division can be made. Accordingly, males express “well-being”, while women can be divided by home typology; if they live in detached houses, the same sensation as males is expressed. Conversely, if women spend their lives in more crowded houses, their sensation modifies to neutral, showing a worsening in rating. This demonstrate how home typology and gender may influence perception;
- the preference to maintain the quieter situation in the future slightly depends on home typology. Most of respondents expressed a desire for a much quieter place. This was particularly stressed by those living in city centres;
- indoor noise is perceived differently by different ages. Accordingly, younger people (< 60 years old) gave a wide range of responses, older respondents (> 60 years old) assessed that indoor noise was quieter;
- age differences were also associated to preferences given about the indoor acoustic situation during the lockdown. Younger respondents (< 40 years old) preferred noisier places, while older people (between 40 and 60 years old) showed less preference for this. When moving to over 60 year-olds, respondents did not want to change anything. This demonstrates how, when rating indoor noise, age plays the most important role. In contrast, home typology and location does not seem to be significant.
